# Optimizing Tissue Sampling Timing for Accurate Gene Expression Analysis

**DOI:** 10.3390/ijms26178581

**Published:** 2025-09-03

**Authors:** Sabina Davidsson, Tomas Jerlström, Jessica Carlsson

**Affiliations:** Department of Urology, Faculty of Medicine and Health, Örebro University, 703 62 Örebro, Sweden; sabina.davidsson@regionorebrolan.se (S.D.); tomas.jerlstrom@regionorebrolan.se (T.J.)

**Keywords:** urinary bladder, surgery, tissue sampling timing, gene expression

## Abstract

The reliability of molecular diagnostic and prognostic tools is contingent on the quality of biospecimens, which are often collected during surgical procedures. This study investigated the impact of surgical manipulation on gene expression in the urinary bladder mucosa during radical cystectomy. Seventeen patients with urinary bladder cancer were enrolled, and paired pre- and post-surgery biopsies were analyzed. Pre-surgical biopsies were obtained in situ under anesthesia, while post-surgical biopsies were collected ex vivo following bladder removal. Total RNA was extracted, and gene expression was assessed using qPCR arrays, measuring the expression of 374 inflammation-related genes. The findings from the exploratory phase were further validated by analyzing key genes in an independent patient cohort using TaqMan^®^ gene-specific assays. Exploratory analysis revealed significant differential expression in 27 genes, with key genes such as IL6, FOS, and PTGS2 being upregulated post-surgery. Validation of five selected genes in an independent cohort confirmed these findings. This study reinforces the necessity of accounting for surgery-induced alterations in gene expression when analyzing tissue samples collected intraoperatively. By elucidating the molecular impact of surgical interventions, this work provides critical insights for refining experimental methodologies and enhancing the interpretability of gene expression studies in clinical and research settings.

## 1. Introduction

The development of molecular diagnostic and prognostic tools is dependent on high-throughput molecular analyses of biospecimens [1]. Each year, millions of biospecimens are collected for research purposes, encompassing a diverse array of tissues and fluids from both healthy individuals and patients with various medical conditions [2]. However, the quality of these biospecimens is paramount, as poor-quality samples can result in inaccurate data interpretation and undermine the reliability of the subsequent analyses.

The quality of biospecimens is closely linked to pre-analytical conditions, encompassing a range of factors from collection techniques to storage protocols [3,4]. For example, the method of acquisition can significantly impact both gene and protein expression. Factors like ischemia time, tissue handling, and exposure to environmental contaminants during collection can all influence the molecular composition of the specimen [5,6,7]. Therefore, meticulous attention to detail during the collection is essential to ensuring biospecimen integrity and the accuracy of downstream analyses.

Biospecimens collected during surgical procedures present unique challenges due to dynamic physiological changes. In addition to ischemia and mechanical tissue manipulation, patients are often exposed to pharmacological agents such as anesthetics and analgesics, which can significantly alter gene expression patterns [6,8]. Understanding the impact of these factors is crucial for accurate interpretation of molecular data from surgical samples.

While previous studies have explored the effects of surgery on gene expression in tissues such as the prostate, breast, diaphragm, and lung [9,10,11,12], the specific impact of surgical manipulation on urinary bladder tissue remains largely unexplored. This study aims to address this gap by investigating whether surgical removal of the urinary bladder induces significant changes in gene expression profiles.

The findings of this study hold significant implications for the design of research studies utilizing gene expression as a biomarker for disease diagnosis, prognosis, and treatment response. By clarifying molecular alterations associated with surgical interventions, researchers can refine experimental methodologies, enhance analytical accuracy, and deepen their understanding of disease pathogenesis and therapeutic outcomes.

## 2. Results

The mean age of the study participants was 67 years (standard deviation (sd): 10.1 years), with a median surgery duration of 300 min (range: 206–735 min) and a median blood loss of 400 milliliters (range: 100–2000 mL). Total RNA samples extracted from both pre- and post-surgery specimens underwent RNA quality assessment. The median RNA Integrity Number (RIN) was 9.0 for both pre- and post-surgery samples, with ranges of 7.3–9.9 and 2.3–10, respectively. No statistically significant difference in RNA quality was observed between pre- and post-surgery samples (*p* > 0.05).

### 2.1. Exploratory Dataset Analysis: Uncovering Insights into Gene Expression Changes

In the initial exploratory phase of this study, 14 samples from pre- and post-surgery specimens of seven patients (*n* = 14) were analyzed using a qPCR array targeting 374 inflammation-associated genes. Of these, 356 genes were expressed in the mucosal membrane of the urinary bladder, with mean C_T_ values greater than 35 in either pre- or post-surgery samples.

**Figure 1:**
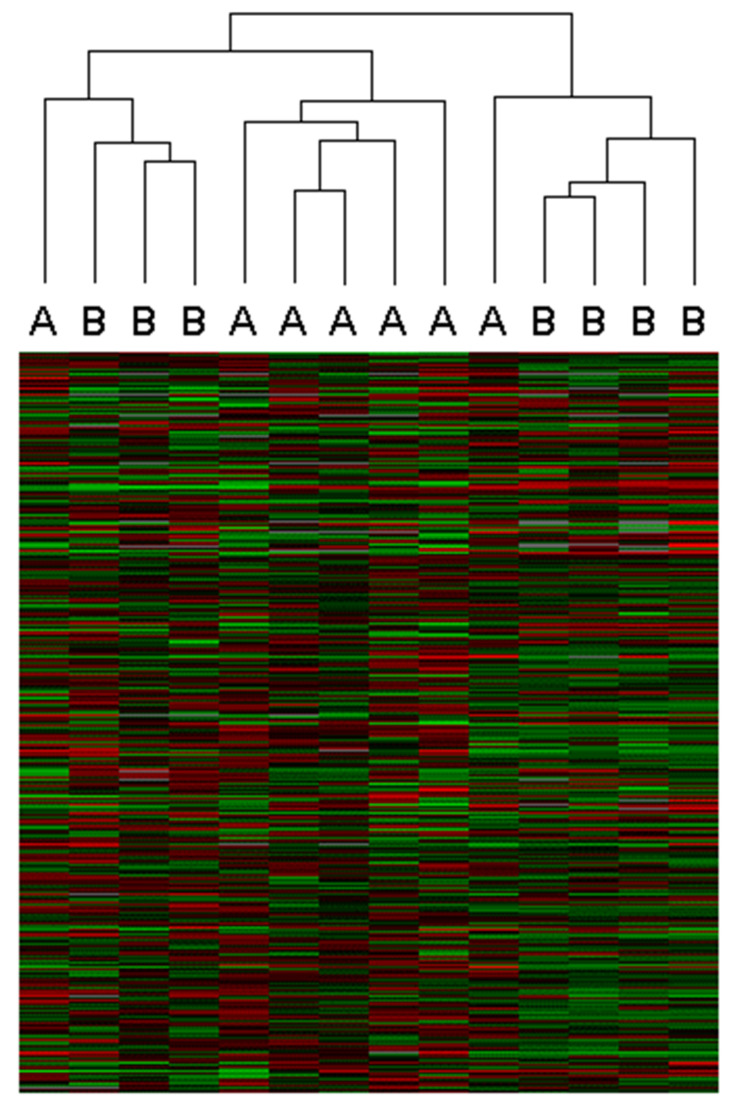
Hierarchical clustering of pre- and post-surgery bladder tissue samples. Unsupervised hierarchical clustering based on the expression of 374 inflammation-associated genes revealed three distinct groups: one containing only post-surgery samples (*n* = 5), and two mixed clusters with varying proportions of pre- and post-surgery specimens. A = post-surgery sample; B = pre-surgery sample.

Hierarchical clustering of all samples and expressed genes revealed three distinct clusters: one exclusively containing post-surgery samples (*n* = 5), another comprising three pre-surgery samples and one post-surgery sample, and a third containing four pre-surgery samples and one post-surgery sample (Figure 1). These results suggest discernible differences in overall gene expression levels between pre- and post-surgery samples.

A paired Student’s t-test identified 106 genes (29.6%) as differentially expressed between pre- and post-surgery samples (*p* < 0.05) (Table S1). Following correction for multiple testing, 27 genes (7.6%) remained significantly differentially expressed (corrected *p* < 0.05). Among these, 14 genes (51.9%) were upregulated, and 13 (48.1%) were downregulated in post-surgery samples compared to pre-surgery samples (Table 1).

### 2.2. Validation Dataset Analysis: Confirming Gene Expression Patterns

To validate the findings from the exploratory phase, a separate set of pre- and post-surgery samples from ten patients was analyzed. This validation focused on the five most differentially expressed genes identified in the exploratory analysis (corrected *p* < 0.01).

**Figure 2:**
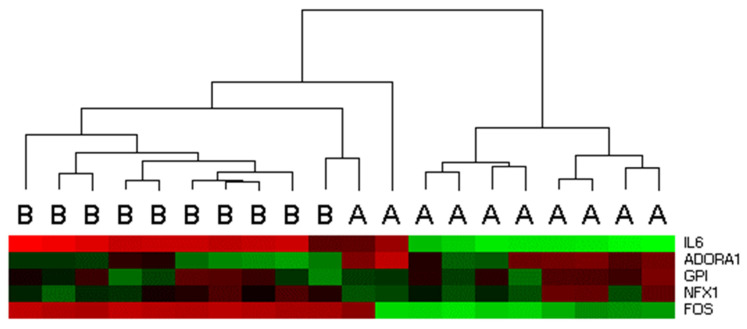
Hierarchical clustering of five differentially expressed genes across 10 bladder tissue samples. Unsupervised hierarchical clustering revealed three clusters: one containing all pre-surgery samples and one post-surgery sample, a second cluster with the remaining eight post-surgery samples, and a third cluster comprising a single post-surgery sample. A = post-surgery sample; B = pre-surgery sample.

Among these genes, IL6 and FOS exhibited consistent upregulation in the validation set, with median fold changes of 125.7 and 34.6, respectively. ADORA1 was downregulated in nine out of ten samples, while GPI and NFX1 were upregulated in six out of ten samples (Table 2). Hierarchical clustering of these five genes revealed three distinct clusters: one comprising all pre-surgery samples and one post-surgery sample, another containing the remaining eight post-surgery samples, and a third with a single post-surgery sample (Figure 2).

## 3. Discussion

This study aimed to evaluate the impact of surgical manipulation during radical cystectomy on gene expression in urinary bladder tissue. The results revealed significant alterations throughout the procedure, affecting approximately 8% of the transcripts investigated.

Factors such as hypoxia, stress, and mechanical tissue manipulation during surgery are believed to influence gene expression [5,6,7,8]. As a result, gene expression studies using tissue collected during surgery may reflect surgery-induced changes rather than solely the tumor’s molecular phenotype. Notably, the nature and timing of tissue sampling during surgery are often inadequately described in the literature, making it difficult to assess the impact of surgical manipulation of gene expression. Greater attention to these aspects is therefore needed in future research.

RNA integrity is a critical factor in gene expression studies, as it can be compromised by acute tissue hypoxia and stress during surgery [13]. However, our findings demonstrate that RNA extracted from post-surgery tissue retained high integrity, comparable to pre-surgery samples. This indicates that the observed changes in gene expression are unlikely to result from RNA degradation.

Previous studies have investigated the impact of surgery on gene expression in tissues, such as the prostate and breast. Although the present study focused on a limited set of genes associated with inflammation and immune pathways, there were overlaps with previous findings. Pedersen et al. reported upregulation of immune pathways in breast tissue following surgical manipulation, with CXCL-2 among the most upregulated genes [10]. Similarly, in the present study, CXCL-2 was one of the most upregulated genes in post-surgery samples, although it was the only overlapping gene between the two studies. Additionally, Lin et al. demonstrated upregulation of several acute-phase response genes in post-surgery prostate samples, including PTGS2 [9]. In alignment with this, PTGS2 was also upregulated in our post-surgery samples. Huang et al. compared gene expression between pre- and post-surgery diaphragm samples, revealing alterations in inflammatory and stress-response genes, including PTGS2, IL6, and FOS [11]. These same genes were affected by surgical manipulation in our study. Despite the limited number of genes investigated in the present study, the overlaps with previous studies reinforce that surgical manipulation during radical cystectomy significantly influences gene expression in urinary bladder tissue.

Several factors during surgery, such as general anesthesia, warm ischemia time, and mechanical tissue manipulation, can influence gene expression in urinary bladder mucosa. In this study, we observed significant alterations in gene expression solely due to surgical excision. Despite the small sample size, substantial differences were identified between pre- and post-surgery samples. However, it is challenging to pinpoint the exact contribution of variables such as warm ischemia or anesthetics to these changes. Previous studies have indicated that the time between surgical resection and tissue stabilization can impact RNA quality and gene expression [5,14]. A strength of this study is that tissue was promptly stabilized in RNAlater after resection. RNAlater rapidly permeates the tissue to stabilize and protect it, obviating the need for immediate processing or freezing. Numerous studies indicate that RNAlater is as effective as, or superior to, snap-freezing in preserving tissue [15,16,17]. Despite these precautions, the precise mechanisms underlying the observed gene expression changes remain unclear.

Several mechanisms may explain the observed changes in inflammation-associated gene expression following surgical manipulation. Prolonged tissue hypoxia during ischemia activates stress-related pathways, including apoptosis, inflammation, and immune signaling [18]. Mechanical strain from surgical manipulation similarly triggers these pathways, increasing expression of mediators such as iNOS and IL-6 and promoting pro-inflammatory differentiation of myeloid cells [19,20]. Anesthetic agents can further modulate immune function by altering cytokine production and suppressing innate and adaptive responses [21,22]. Although this study was not designed to determine the relative contribution of each factor, these represent plausible drivers of the observed gene expression changes. Future studies should systemically record ischemia times and anesthetic regimens, include multiple biopsies per patient, and expand the gene panel or perform whole-transcriptome analyses. Such approaches will help clarify the relative contributions of hypoxia, mechanical stress, and anesthetic exposure, improving the interpretation of gene expression in surgical tissue samples.

Several limitations of our study must be acknowledged. First, individual ischemia times and anesthetic regimens were not recorded. Both factors are known to influence gene expression and may have contributed to variability in our findings. As adjustments for these variables were not possible, the observed changes should be interpreted with this potential confounding in mind. Additionally, only 374 unique genes were investigated. Had a broader range of genes been examined, the number of differentially expressed genes between pre- and post-surgery samples would likely have been greater. This assumption is supported by other studies investigating the impact of surgery on gene expression levels. Future research should, therefore, analyze a wider gene set or apply whole-transcriptome approaches to more comprehensively capture the molecular effects of surgical manipulation. Finally, analyzing only one tissue biopsy per patient at a single time point precluded investigation into whether differential gene expression was attributable to tissue heterogeneity.

## 4. Materials and Methods

### 4.1. Patient Samples

**Figure 3:**
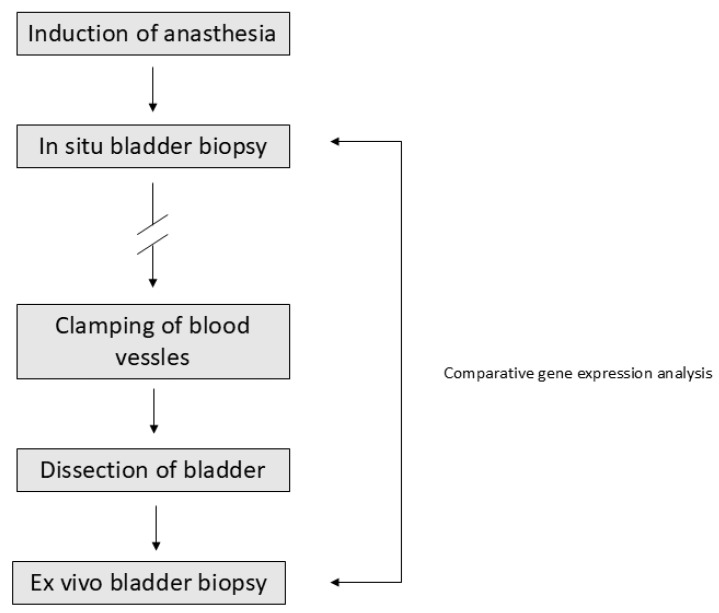
Study design and sample collection workflow. Patients with urinary bladder cancer scheduled for radical cystectomy were included. Following induction of anesthesia, four in situ biopsies were collected from macroscopically normal bladder mucosa (pre-surgery). After clamping of blood vessels and bladder dissection, four ex vivo biopsies were obtained from the same region (post-surgery).

Seventeen patients diagnosed with urinary bladder cancer (UBC) and scheduled for radical cystectomy at the Department of Urology, University Hospital of Örebro, Sweden, between 2013 and 2016 were included in this study. After induction of anesthesia, four cold in situ biopsies were obtained from macroscopically normal mucosa within the urinary bladder (pre-surgery). Following bladder removal, another four cold ex vivo biopsies were obtained from the same area of the mucosa (post-surgery). The study design is summarized in Figure 3.

All biopsies were promptly placed in RNAlater RNA stabilization reagent (Qiagen, Hilden, Germany) and stored at −80 °C until further analysis. Tissue displaying macroscopic normal histology was selected to minimize tissue heterogeneity compared to tumor tissue. The study was approved by the Swedish ethical review authority and the ethical review board in the Uppsala and Örebro region (Approval number: 2012/186). All participants provided informed consent. The study adhered to the Declaration of Helsinki.

### 4.2. Gene Expression Analysis

RNA extraction from the biopsies was performed using the AllPrep^®^ DNA/RNA/protein kit combined with the RNeasy^®^ MiniElute^®^ Cleanup Kit (Qiagen, Hilden, Germany). The AllPrep^®^ protocol was followed up to step 14, after which the RNeasy^®^ protocol was applied, as outlined in Qiagen’s supplementary protocol for purification of miRNAs from cells and tissues using the AllPrep DNA/RNA/protein kit.

Total RNA concentration (ng/µL) was quantified using a NanoDrop ND-100 spectrophotometer (ThermoFisher Scientific, Waltham, MA, USA), and RNA purity was assessed by the A260/A280 ratio. RNA quality was further evaluated using the 2100 BioAnalyzer system with the RNA 6000 Pico Kit (Agilent Technologies, Santa Clara, CA, USA).

#### 4.2.1. Explorative Dataset

The SuperScript^®^ VILO^TM^ Mastermix (ThermoFisher Scientific, Waltham, MA, USA) was used to convert 10 ng of RNA into cDNA. Pre-amplification was performed using Custom TaqMan^®^ PreAmp Pools and TaqMan^®^ PreAmp Master Mix according to the Custom TaqMan^®^ PreAmp Pools Protocol (ThermoFisher Scientific, Waltham, MA, USA).

Following pre-amplification, the cDNA was combined with Custom TaqMan^®^ Primer Pools and TaqMan^®^ PCR master mix (No AmpErase UNG, ThermoFisher Scientific, Waltham, MA, USA) and subjected to a 40-cycle qPCR reaction on custom TaqMan^®^ low-density arrays. These arrays were specifically designed to measure the expression of 374 inflammation-associated genes and six endogenous control genes (Table S2). All reactions were performed using the Applied Biosystems 7900 HT System.

#### 4.2.2. Validation Dataset

The SuperScript^®^ VILO^TM^ Mastermix (ThermoFisher Scientific, Waltham, MA, USA) was used to convert 10 ng of RNA into cDNA. Quantification was performed using gene-specific TaqMan probes for five target genes and two endogenous controls (Table S3) with TaqMan^®^ PCR master mix (No AmpErase UNG, ThermoFisher Scientific, Waltham, MA, USA). Reactions were conducted on the Applied Biosystems 7900 HT System.

### 4.3. Statistical Analyses

The exploration dataset was normalized using qPCRNorm quantile normalization. The stability of the endogenous control genes on the TaqMan^®^ low-density arrays was assessed using the NormFinder v1.0 and BestKeeper v1.0 software [23,24].

The validation dataset was normalized using the geometric mean of the two most stable control genes identified in the exploration dataset (18S5 and GUSB). Differential expression between pre- and post-surgery samples was evaluated using a paired Student’s t-test. To account for multiple testing, the Benjamini–Hochberg method was applied to adjust *p*-values in the exploration dataset. Statistical significance was considered at *p* < 0.05.

All statistical analyses were performed using the R programming software v4.0.5 [25]. Hierarchical clustering analysis was executed using the PermutMatrix clustering tool v1.9.3 [26], utilizing the Euclidean distance for measuring similarity between expression profiles and employing the average linkage rule for clustering.

## 5. Conclusions

The findings of this study highlight the significant impact of surgical manipulation on gene expression. It is important to recognize that changes observed in biospecimens obtained through surgical resection may not exclusively reflect disease severity or treatment response; rather, they could also be influenced by the method of tissue acquisition.

Our results emphasize the importance of carefully considering the acquisition of biospecimens when designing and interpreting gene expression studies for clinical applications. Understanding the potential confounding effects of surgical manipulation is crucial for accurately interpreting study results and drawing meaningful conclusions about disease biology and treatment efficacy.

Future studies should, therefore, (1) record warm ischemia times and anesthetic regimens systematically, (2) apply broader gene panels or whole-transcriptome approaches to capture the full spectrum of molecular alterations, and (3) include multiple tissue biopsies or sampling time points to account for intra-tissue heterogeneity. Such methodological refinements will enable more precise attribution of gene expression changes to either disease-related biology or surgical effects, thereby improving the reliability of molecular biomarkers in clinical research.

## Figures and Tables

**Table 1:** Differentially expressed genes between seven paired pre- and post-surgery samples.

Gene	Raw *p*-Value	Corrected *p*-Value ^a^	FC ^b^
FOS	7.73 × 10^−7^	0.0002	32.71
NFX1	1.89 × 10^−5^	0.0033	0.49
GPI	4.57 × 10^−5^	0.0049	0.43
IL6	5.56 × 10^−5^	0.0049	73.57
ADORA1	9.08 × 10^−5^	0.0065	0.21
OSM	0.0002	0.012	13.25
CXCL2	0.0005	0.026	26.31
VEGFA	0.0006	0.028	4.30
IFNA2	0.0007	0.028	5.11
IL10	0.0007	0.028	2.53
IL2RG	0.001	0.042	0.41
IL17RB	0.001	0.042	0.32
TNFSF15	0.002	0.046	0.26
IFNA14	0.002	0.049	6.07
FASLG	0.002	0.049	0.18
NFATC3	0.002	0.049	0.40
CEBPB	0.003	0.049	4.31
INHBB	0.003	0.049	2.86
IL23R	0.003	0.049	0.41
NAMPT	0.003	0.049	3.65
PTGS2	0.003	0.049	21.12
F3	0.003	0.049	4.70
CD70	0.004	0.049	0.45
IL13RA1	0.004	0.049	0.56
IFNAR1	0.004	0.049	0.55
IRF4	0.004	0.049	2.02
PARP4	0.004	0.049	0.41

^a^ Benjamini–Hochberg corrected *p*-value. ^b^ Median fold change.

**Table 2:** Validation of five genes in a dataset of ten pre- and post-surgery samples.

Gene	FC ^a^	Up/Down	No. of Patients	*p*-Value	Corrected *p*-Value ^b^
IL6				5.6 × 10^−5^	0.005
	125.7	↑	10		
ADORA1				9.1 × 10^−5^	0.006
	1.8	↑	1		
	0.4	↓	9		
GPI				4.6× 10^−5^	0.005
	1.4	↑	6		
	0.6	↓	4		
NFX1				1.9 × 10^−5^	0.003
	1.3	↑	6		
	0.6	↓	4		
FOS				7.7 × 10^−7^	0.0003
	34.6	↑	10		

^a^ Median fold change. ^b^ Benjamini–Hochberg corrected *p*-value. ↑ = Up-regulation ↓ = Down-regulation

## Data Availability

The data are available upon request due to ethical restrictions.

## Supplementary Materials

The following supporting information can be downloaded at https://www.mdpi.com/article/10.3390/ijms26178581/s1.

## Abbreviations

The following abbreviations are used in this manuscript:

FC	Fold Change
RIN	RNA Integrity Number
sd	Standard Deviation
UBC	Urinary Bladder Cancer
